# A new species of *Poemenia* Holmgren with a key to species known from China and the Eastern Palaearctic Region (Hymenoptera, Ichneumonidae, Poemeniinae)

**DOI:** 10.3897/zookeys.653.11672

**Published:** 2017-02-08

**Authors:** Shu-Ping Sun, Mao-Ling Sheng, Tian-Lin Chen

**Affiliations:** 1 General Station of Forest Pest Management, State Forestry Administration, 58 Huanghe North Street, Shenyang 110034, P.R. China; 2 Forest Protection Station of Haicheng, Haicheng, Liaoning 114200, P.R. China

**Keywords:** Key, new species, *Poemenia*, Poemeniinae, *Quercus wutaishanica*, taxonomy

## Abstract

A new species of Poemeniinae, *Poemenia quercusia* Sun & Sheng, **sp. n.**, is described and illustrated. Specimens were reared from twigs of *Quercus wutaishanica* Blume in Haicheng, Liaoning province, P.R. China. A key to species known from China and the Eastern Palaearctic Region is provided.

## Introduction

*Poemenia* Holmgren, 1859, belonging to the tribe Poemeniini of the subfamily Poemeniinae (Hymenoptera: Ichneumonidae), comprises 16 species (Sheng et al. 2016, Yu et al. 2016), of which three are from the Oriental Region (Gupta 1980), seven from the Eastern Palaearctic Region (two of them are found across the Palaearctic), four from the Western Palaearctic (Haupt 1917, 1938, Yu et al. 2016), and four from the Nearctic Region (Yu et al. 2016). Eight species of *Poemenia* Holmgren were known from China until now (He et al. 1996, Sheng and Sun 2010, 2016, Sonan 1936). The diagnostic characters of the genus were elucidated by Townes (1970) and expanded upon by Gupta (1980).

The hosts of *Poemenia* Holmgren mainly belong to Crabronidae (Hymenoptera) (Shaw 2006). Some hosts, belonging to Cerambycidae, Lamiidae, Sphecidae, also were reported (Jussila and Kapyla 1975, Kusigemati 1986, Uhthoff-Kaufmann 1990, 1991, Yu et al. 2016).

In the last two years the authors have been exploring mountains in Haicheng, Liaoning Province, situated near the southern border of the Eastern Palaearctic part of China, and gathered many wood and tree branches infested by woodborers, and have collected large numbers of ichneumonids. In this article a new species of *Poemenia* is described. The species was reared from twigs of *Quercus wutaishanica* Blume.

The type locality is a forest comprised of mixed deciduous angiosperms and evergreen conifers, mainly including *Quercus wutaishanica*, *Quercus* sp., *Larix* sp., *Castanea* spp. and *Pinus tabulaeformis* Carr.

## Material and methods

Rearing parasitoids. Trunks and twigs of naturally infested *Quercus wutaishanica* trees were brought to the laboratory and maintained in a large nylon cage at room temperature. Water was sprayed over the trunks and twigs twice a week and emerged insects collected daily.

Images were taken using a Leica M205A Stereomicroscope with LAS Montage MultiFocus. Morphological terminology is mostly based on Gauld (1991). Specimens of *Poemenia brachyura* Holmgren, 1860 and *Poemenia hectica* (Gravenhorst, 1829), preserved in the Natural History Museum, London, UK (BMNH), were checked.

Type specimens are deposited in the Insect Museum, General Station of Forest Pest Management (GSFPM), State Forestry Administration, People’s Republic of China.

## Results

### Poemenia

Taxon classificationAnimaliaHymenopteraIchneumonidae

Holmgren, 1859

Poemenia Holmgren, 1859: 130. Type-species. *Poemenia notata* Holmgren.

#### Diagnosis.

Interior margins of eyes ventrally subparallel. Clypeus approximately 2.0 times as wide as long, evenly convex. Mandible with two teeth, lower tooth rather elongate. Upper portion of gena not or finely sculptured. Mesoscutum without transverse wrinkles or with fine wrinkles associated with notauli. Epicnemial carina absent. Areolet present or absent. Hind wing vein 1-cu distinctly shorter than cu-a. Claw simple. Second tergite without anterolateral grooves.

#### Key to species of *Poemenia* from China and the Eastern Palaearctic Region

**Table:** 

1	First tergite 1.5 times as long as apical width. Second to fourth tergites each with a rhombic depression. Fore wing with large areolet. Large spots on mesoscutum and metapleuron reddish yellow	***Poemenia depressa* Wang & Gupta**
–	First tergite 2.5 times or more as long as apical width. Second to fourth tergites without rhombic depressions. Fore wing without or with relatively small areolet. Mesoscutum and metapleuron entirely black	**2**
2	Fore wing without areolet	**3**
–	Fore wing with areolet	**4**
3	Face with dense long opalescent hairs (Fig. 8). First tergite 2.7 times as long as apical width. Third tergite shorter than second tergite. Basal halves of hind tarsomeres 1 to 3 and base of tarsomere 4 white	***Poemenia quercusia* Sun & Sheng, sp. n.**
–	Face with dense punctures. First tergite 3.0 times as long as apical width. Third tergite as long as second tergite. Hind tarsomeres entirely black	***Poemenia taiwana* Sonan**
4	Mesopleuron and mesosternum reddish-brown	**5**
–	Mesopleuron and mesosternum black	**6**
5	First tergite approximately 3.6 times as long as apical width, 1.3 times as long as second tergite. First sternite (Fig. 3) extending to 0.7 length of tergite, spiracle located at basal 0.3 of tergite. Third tergite approximately 1.9 as long as apical width. Ovipositor sheath 0.8 times as long as fore wing, 0.9 as long as metasoma. Hind coxa reddish-brown, with basal white spot	***Poemenia maculata* Sheng & Sun**
–	First tergite approximately 2.9 times as long as apical width, 1.1 times as long as second tergite. First sternite (Fig. 2) extending to 0.5 length of tergite, spiracle located slightly basal mid of tergite. Third tergite approximately 1.4 as long as apical width. Ovipositor sheath 0.5 times as long as fore wing, 0.45 as long as metasoma. Hind coxa entirely reddish-brown	***Poemenia brevis* Sheng & Sun**
6	Ovipositor sheath 0.4 to 0.5 times as long as fore wing. Face with fine, indistinct punctures. Lower portion of mesopleuron and propodeum with distinct punctures.	***Poemenia brachyura* Holmgren**
–	Ovipositor sheath at least as long as fore wing. Face with distinct punctures. Sculpture not entirely as above, or lower portion of mesopleuron with indistinct punctures, or propodeum with rugae	**7**
7	Lateral portion of pronotum with strong transverse ridge. Areolet uniquely small, 3rs-m approximately 2.8 times as long as 2rs-m, receiving vein 2m-cu at lower posterior corner	***Poemenia qinghaiensis* Sheng & Sun**
–	Pronotum normal, without transverse ridge. Areolet relatively large, vein 3rs-m at most 2.0 times as long as 2rs-m, receiving 2m-cu distinctly mesad of posterior corner	**8**
8	Areolet receiving vein 2m-cu approximately 0.7 times distance from vein 2rs-m to 3rs-m. Propodeum with fine transverse rugae. Ovipositor sheath 1.1 to 1.2 times as long as fore wing. Anterolateral portion of pronotum yellow	***Poemenia hectica* (Gravenhorst)**
–	Areolet receiving vein 2m-cu at or slightly mesad of lower posterior corner. Propodeum without rugae. Ovipositor sheath approximately 0.9 to 1.0 times as long as fore wing. Pronotum entirely black	***Poemenia pedunculata* He**

**Figures 1–6.**
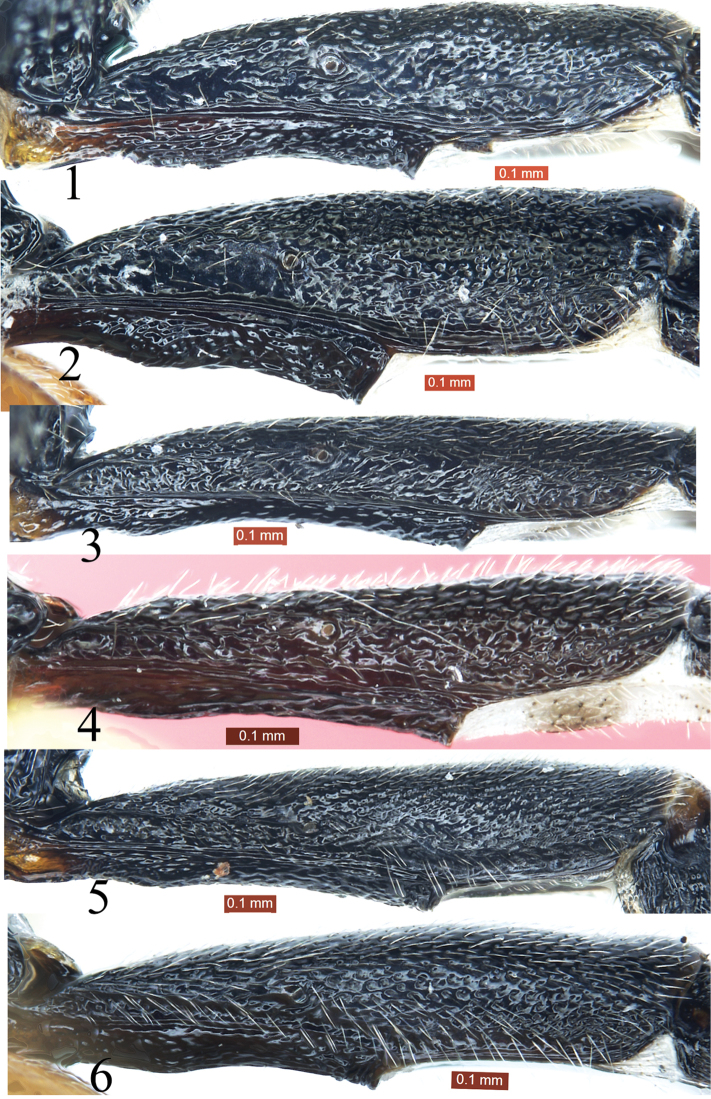
First tergites, lateral view. **1** *Poemenia brachyura* Holmgren, 1860 **2** *Poemenia brevis* Sheng & Sun, 2010 **3** *Poemenia maculata* Sheng & Sun, 2016 **4** *Poemenia qinghaiensis* Sheng & Sun, 2016 **5** *Poemenia pedunculata* He, 1996 **6** *Poemenia quercusia* Sun & Sheng, sp. n.

### Poemenia quercusia

Taxon classificationAnimaliaHymenopteraIchneumonidae

Sun & Sheng sp. n.

http://zoobank.org/D9334CD9-4E8C-4207-8508-8596F57D1A20

Figures 7–11 , 12–14

#### Etymology.

The specific name is derived from the name of the plant the specimens were reared from.

#### Material examined.

Holotype. Female, Chagou, Haicheng, Liaoning, 18 May 2015, Mao-Ling Sheng. Paratype. 1 male, Chagou, haicheng, Liaoning, 6 July 2015, Mao-Ling Sheng.

#### Diagnosis.

Face (Fig. 8) with dense long opalescent hairs. Hind wing vein 1-cu almost vertical, nearly 0.3 times as long as cu-a; cu-a strongly reclivous. Propodeum (Fig. 11) evenly longitudinally convex, without carinae, with dense opalescent hairs. Propodeal spiracle circular. First sternite extending to 0.5 length of tergite. Second tergite approximately 1.5 times as long as apical width. Hind coxa and femur red brown, basal portions of tarsomeres 1 to 4 white.

**Figures 7–11.**
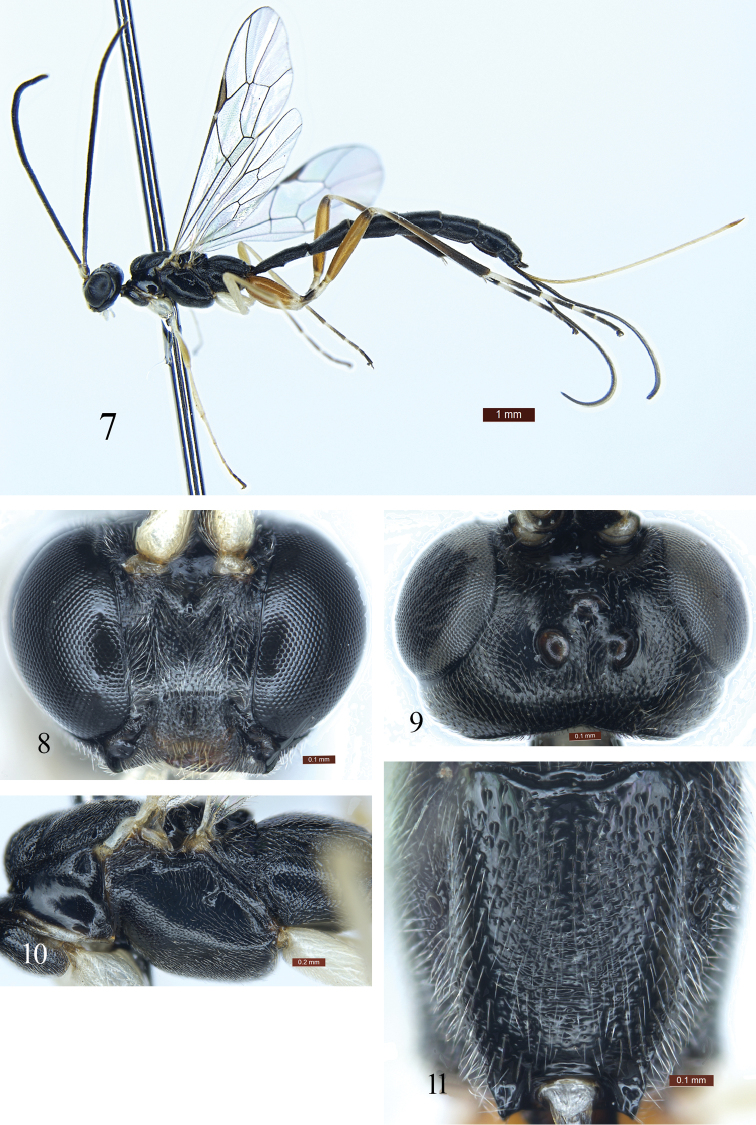
*Poemenia quercusia* Sun & Sheng, sp. n. Holotype. Female. **7** Habitus, lateral view **8** Head, anterior view **9** Head, dorsal view **10** Mesosoma, lateral view **11** Propodeum.

#### Description.

Female. Body length approximately 8.5 mm. Fore wing length approximately 5.5 mm. Ovipositor sheath length approximately 4.5mm.

*Head*. Inner margins of eyes slightly convergent ventrally. Face (Fig. 8) with dense long opalescent hairs; upper margin with a small median tubercle. Clypeus 2 times as wide as long, with dense fine punctures; apical margin slightly concave, with dense light brown hairs. Mandible with dense fine punctures. Malar space approximately 0.2 times as long as basal width of mandible. Gena in lateral view 0.5 times as long as width of eye, with even, fine punctures. Vertex (Fig. 9) with dense indistinct punctures. Postocellar line approximately 0.86 times as long as ocular-ocellar line. Frons with fine punctures, median longitudinal portion shiny. Antenna with 31 flagellomeres.

*Mesosoma*. Pronotum smooth, shiny; upper posterior portion with distinct fine punctures. Epomia weak. Mesoscutum with dense indistinct fine punctures; median portion slightly concave, with irregular short rugae. Scutellum with fine punctures. Mesopleuron (Fig. 10) with fine uneven punctures. Mesopleural fovea consisting of shallow horizontal groove connecting with mesopleural suture. Upper anterior portion of metapleuron with shallow punctures, lower posterior portion with oblique longitudinal rugae. Wings slightly brownish, hyaline. Fore wing with vein 1cu-a opposite 1/M. Areolet absent. Distance from vein 2rs-m to 2m-cu approximately 1.2 times as long as 2rs-m. 2-Cu approximately as long as 2cu-a. Hind wing vein 1-cu almost vertical, approximately 0.3 times as long as cu-a; cu-a strongly reclivous. Ratio of length of hind tarsomeres 1:2:3:4:5 is 10.0:4.0:2.6:1.7:2.0. Propodeum (Fig. 11) evenly longitudinally convex, without carinae, with dense opalescent hairs; apical-median portion with indistinct transverse rugae. Propodeal spiracle circular.

**Figures 12–14.**
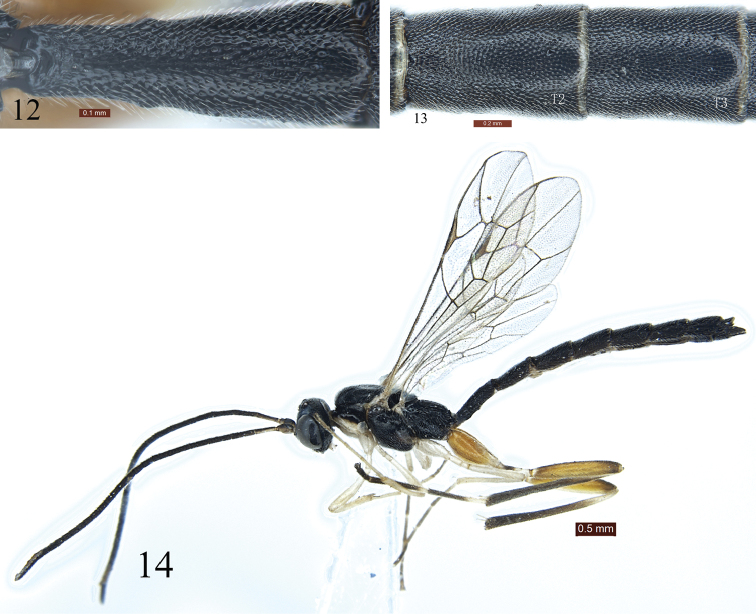
*Poemenia quercusia* Sun & Sheng, sp. n. Holotype. Female. **12** First tergite, dorsal view **13** Tergites 2 and 3, dorsal view **14** Paratype, male, habitus, lateral view.

*Metasoma*. First tergite (Figs 6, 12) approximately 2.7 times as long as apical width, 1.3 times as long as second tergite (Fig. 13), subcylindrical; with dense indistinct punctures and shallow median longitudinal groove; spiracle located at basal 0.38 of first tergite. First sternite extending to 0.5 length of tergite. Tergites 2 to 6 with dense punctures. Second tergite approximately 1.5 times as long as apical width. Tergites 3 to 6 parallel-sided. Third tergite approximately 1.4 times as long as apical width. Ovipositor sheath 0.88 times as long as fore wing, 0.9 times as length of metasoma.

*Color* (Fig. 7). Black, except following. Maxillary and labial palpi, ventral profiles of scape and pedicel, anterior margin of pronotum, tegula, fore and mid coxae and trochanters white. Basal-dorsal profile of flagellum slightly yellowish brown. Fore and mid legs yellowish white, dorsal profiles slightly yellowish brown. Tarsomeres 4 and 5 of fore tarsus dark brown. Apical portion of tarsomeres 1 to 3, and entire 4 and 5 of mid tarsus brownish black. Hind coxa and femur red brown. Apex of hind femur black. Apex of hind coxa and trochantellus mainly, basal end of femur, basal portions of tibia, basal halves of tarsomeres 1 to 3 and base of tarsomere 4 white. Pterostigma and veins brown.

**Male** (Fig. 14). Body length approximately 6.8 mm. Fore wing length approximately 4.2 mm. Antenna with 26 flagellomeres.

#### Host.

Unknown, but reared from twigs of *Quercus wutaishanica* Blume. Many specimens of *Carcilia* sp. (Coleoptera: Curculionidae) were also reared from these twigs. But confirmed hosts of *Poemenia* are solitary aculeate wasps such as *Passaloecus* species (Hymenoptera: Crabronidae) (e.g. Shaw 2006) and it is possible that the hosts of *Poemenia quercusia* sp. n. were nesting in beetle burrows in these twigs.

#### Remarks.

This new species is similar to *Poemenia taiwana* Sonan, 1936, but can be distinguished from the latter by the following combinations of characters: apical margin of clypeus evenly concave, with dense light brown hairs; first tergite approximately 2.7 times as long as apical width; third tergite distinctly shorter than second tergite; hind coxa reddish brown; basal halves of tarsomeres 1 to 3 and base of tarsomere 4 white. *Poemenia taiwana*: apical margin of clypeus rounded, without dense light brown hairs; first tergite nearly 3.0 times as long as apical width; third tergite as long as second tergite; hind coxa with only ventral profile reddish-brown; tarsomeres entirely black. They can be distinguished by the key provided above.

## Supplementary Material

XML Treatment for Poemenia

XML Treatment for Poemenia quercusia
